# Bio-Organic Fertilizer: A Green Technology to Reduce Synthetic N and P Fertilizer for Rice Production

**DOI:** 10.3389/fpls.2021.602052

**Published:** 2021-03-23

**Authors:** Umme Aminun Naher, Jatish Chandra Biswas, Md. Maniruzzaman, Faruk Hossain Khan, Md. Imran Ullah Sarkar, Afsana Jahan, Md. Hasibur Rahaman Hera, Md. Belal Hossain, Aminul Islam, Md. Rafiqul Islam, Md. Shahjahan Kabir

**Affiliations:** Bangladesh Rice Research Institute, Gazipur, Bangladesh

**Keywords:** biochar, free-living N_2_ fixing bacteria, kitchen waste, indoleacetic acid, nitrogen use efficiency, phosphate solubilizing bacteria, phosphorus use efficiency

## Abstract

Decomposed organic materials, in combination with plant growth-promoting bacteria (PGPB), are environmentally friendly and reduce synthetic fertilizer use in rice production. A bio-organic fertilizer (BoF) was prepared using kitchen waste (79%), chita-dhan (unfilled rice grain) biochar (15%), rock phosphate (5%), and a consortium of 10 PGPB (1%) to supplement 30% nitrogen and to replace triple superphosphate (TSP) fertilizer in rice production with an improvement of soil health. PGPB were local isolates and identified using 16S ribosomal RNA partial gene sequences as *Bacillus mycoides*, *Proteus* sp., *Bacillus cereus, Bacillus subtilis, Bacillus pumilus, Paenibacillus polymyxa*, and *Paenibacillus* spp. Isolates could fix N_2_ by 0.7–1.4 g kg^–1^, solubilize 0.1–1.2 g kg^–1^ phosphate, and produce 0.1–40 g kg^–1^ indoleacetic acid. The performance of BoF was evaluated by 16 field experiments and 18 farmers’ field demonstration trials during the year 2017–2020 in different parts of Bangladesh. Performances of BoF were evaluated based on control (T_1_), full synthetic fertilizer dose of N, P, and K (T_2_), BoF (2 t ha^–1^) + 70% N as urea + 100% K as muriate of potash (T_3_), 70% N as urea + 100% P as TSP + 100% K as muriate of potash (T_4_), and 2 t ha^–1^ BoF (T_5_) treatments. At the research station, average grain yield improved by 10–13% in T_3_ compared with T_2_ treatment. Depending on seasons, higher agronomic N use efficiency (19–30%), physiological N use efficiency (8–18%), partial factor productivity (PFP)_N_ (114–150%), recovery efficiency (RE)_N_ (3–31%), N harvest index (HI_N_) (14–24%), agronomic P use efficiency (22–25%), partial factor productivity of P (9–12%), ARE_P_ (15–23%), and HI_P_ (3–6%) were obtained in T_3_ compared with T_2_ treatment. Research results were reflected in farmers’ field, and significant (*P* < 0.05) higher plant height, tiller, panicle, grain yield, partial factor productivity of N and P were obtained in the same treatment. Application of BoF improved soil organic carbon by 6–13%, along with an increased number of PGPB as compared with full synthetic fertilizer dose. In conclusion, tested BoF can be considered as a green technology to reduce 30% synthetic N and 100% TSP requirements in rice production with improved soil health.

## Introduction

Rice dictates food security in many Asian countries, such as Bangladesh. Rice cultivation involves a large number of nutrients that are derived from synthetic fertilizers. Among the plant nutrients, nitrogen (N) is the most limiting factor for rice production, and its application rate is higher than the other nutrients. In Bangladesh, rice grows three times in a year, covering 10 m ha of land. Average synthetic N use in the wet and dry seasons is 65 and 100 kg ha^–1^, respectively, which partakes approximately 83% of the total urea-N fertilizer of the country (BBS, 2017). Unfortunately, N use efficiency is poor in rice cultivation, and approximately 50% of applied N is lost to the environment (Islam et al., 2016), in the form of ammonia, nitrate, and nitrogen oxides through volatilization, leaching, and emissions, respectively (Sutton et al., 2013; Battye et al., 2017), causing serious environmental pollution and human health hazards (Schlesinger, 2009; Canfield et al., 2010). Phosphorus (P) is the second most important nutrient and is held naturally in the earth as rock phosphate (RP), which is used as the raw material of synthetic fertilizers such as triple superphosphate (TSP) or other phosphate fertilizer. The solubility of RP limits its use in annual crop production, including rice. Furthermore, any fertilizer production, transportation, and consumption cause greenhouse gas emission, and it is energy involving process. Park et al. (2012) stated that the use of N fertilizer added approximately 20% nitrous oxide (N_2_O) accumulation since the industrial revolution, and approximately 2% of global energy is used for reactive N (Nr) production through the Haber–Bosch process (Sutton et al., 2013). Moreover, approximately 120 Tg N year^–1^ is added into the environment due to synthetic N production (IFA, 2016). Besides chemical synthesis, biological N_2_ fixation (BNF) is another important Nr source. According to Ladha et al. (2016), BNF by symbiotic and free-living microbes add 30–51 Tg Nr to the atmosphere. Wetland rice ecosystem can harbor a diverse group of plant growth-promoting bacteria (PGPB), which efficiently colonizes with a root that has been shown to fix a substantial amount of N_2_ (Hurek et al., 1997; Naher et al., 2013) and solubilize organic and inorganic phosphate (Panhwar et al., 2014). Since the ancient period, *Bacillus*, *Azospirillum*, *Pseudomonas, Rhizobium*, and *Burkholderia* were being used as biofertilizer due to their unique characteristics of N_2_ fixing, phosphate solubilizing, disease suppression, and indoleacetic acid (IAA) production (Pathak and Kumar, 2016; Di Benedetto et al., 2017). Proper management of such bacteria can compensate for synthetic N fertilizer use and promote RP in rice cultivation. Conversely, soil microorganisms play a crucial role in soil nutrient cycling (Richardson et al., 2009; Jacoby et al., 2017) and are known as indicators to maintain soil quality and soil health (Doran and Zeiss, 2000). The number and types of soil microbial inhabitants’ and their activity in the vicinity are crucial to maintaining crop productivity, soil health, and ecosystem functions.

Maintenance of rice soil health is crucial for obtaining national food security in Bangladesh. However, intensive cropping and continuous use of synthetic fertilizers are responsible for reducing soil organic matter (SOM) content, and approximately 83% of cultivated lands are with low SOM (Jahiruddin and Satter, 2010). It had proven that long-term chemical fertilization without applying organic materials impaired soil health and plateaued rice yield, although there were so many improved rice varieties (Naher et al., 2020). The correction of nutrients deficiency by synthetic fertilizers in all kinds of soil is a short-term management strategy. Nevertheless, if we consider soil health, breaking yield ceiling, and sustainable rice production, we need to improve rice soil biochemical properties. Additionally, soil health restoration for higher crop productivity requires external application of SOM and improved soil biology. However, major sources of organic matter in the country (cow dung and poultry litter) are scarce due to its diverse use as fish feed, animal feed, and fuel. In this context, biodegradable kitchen waste can be an alternate option of SOM in densely populated countries, such as Bangladesh. Because of the high population pressure, waste management, especially household wastes (mostly kitchen waste) produced in the urban and semi-urban areas, is a big issue in the country. Parvin and Begum (2018) estimated that the population density was 1,015 people per square kilometer and per capita waste generation was 0.56 kg day^–1^. In many cases, wastes are dumped onto the roadsides, creating an unhealthy environment. Dhaka city alone generates approximately 5,800 tons of solid organic waste each day, at least 80% of which is suitable for composting (Rothenberger et al., 2006). About half of it is collected by Dhaka City Corporation, and the rest remains in open areas and creates environmental pollution. Proper management of such waste reduces greenhouse gas emissions by approximately 89,000 tons of carbon dioxide year^–1^ (Parvin and Begum, 2018). Nonetheless, our preliminary observation indicates that co-composting of these materials with RP improves bioavailable P contents (Naher et al., 2018). Co-composting of household waste materials with RP and phosphate solubilizing bacteria (PSB) may provide a new era of P fertilizer management in rice cultivation and improve rice soil health without the use of any synthetic P fertilizer.

Increasing global demand for food production with population pressure forces intensive agriculture to lean toward synthetic fertilizer use and simultaneously increases risks of soil degradation and environmental pollution by altering the earth’s biogeochemical processes (Singh et al., 2014; Amundson et al., 2015; Steffen et al., 2015). Furthermore, among the agricultural inputs, synthetic fertilizers are required in huge amounts, and every year, the government has to subsidize urea and TSP fertilizers for crop production. Potential management of BNF and PSB may decrease the demand for synthetic N and P fertilizer requirements for rice production. Considering soil health and the environment, we hypothesized that BNF by free-living N_2_ fixing bacteria might compensate at least 30% of Nr in rice production, and co-composting of biodegradable kitchen waste with PR and PSB may fulfill the required P demand of rice and consecutively improve soil health *via* the addition of organic matter. To verify this hypothesis, a bio-organic fertilizer was prepared using biodegradable kitchen waste, RP, chita-dhan biochar, and consortium of locally isolated PGPB and, finally, applied to the rice field. Hence, a study was undertaken with the objectives: (i) to evaluate the efficacy of formulated bio-organic fertilizer that can supplement 30% N and eliminate 100% TSP fertilizer use in rice production and (ii) to find the impact of bio-organic fertilizer application on rice soil biology and carbon restoration as well soil health.

## Materials and Methods

### Formulation of Bio-Organic Fertilizer

Bio-organic fertilizer consists of solid based (20% moisture) organic material (particle size is 2 mm) that was developed at the soil microbiology laboratory of Bangladesh Rice Research Institute (BRRI) Gazipur. Active ingredients of the product are 0.5–1% inoculum mass of locally isolated 10 bacteria (free-living N_2_ fixing, PSB, and IAA producing bacteria) and RP (5%), whereas carrier materials were biodegradable kitchen waste/vegetable waste (79.5%) available in the kacha bazar (local market) and chita-dhan biochar (15%). The bacteria were cultured in 2.5% molasses by maintaining a population of 10^9^ CFU ml^–1^ and added during co-composting of organic materials along with RP and biochar. The product contained approximately 250 g kg^–1^ organic carbon, 10.4 g kg^–1^ N, and approximately 10.4 g kg^–1^ available P, which was sufficient to replace TSP (100%) fertilizer for a single rice crop. It also contained 9.1 g kg^–1^ exchangeable K, 3.5 g kg^–1^ available S, 0.03 mg kg^–1^ available Zn, 0.006 mg kg^–1^ Cu, 0.01 mg kg^–1^ Pb, and 0.67 mg kg^–1^ Cd. The pH of the product was 7.2.

### Isolation and Identification of the Growth-Promoting Bacteria

**FIGURE 1:**
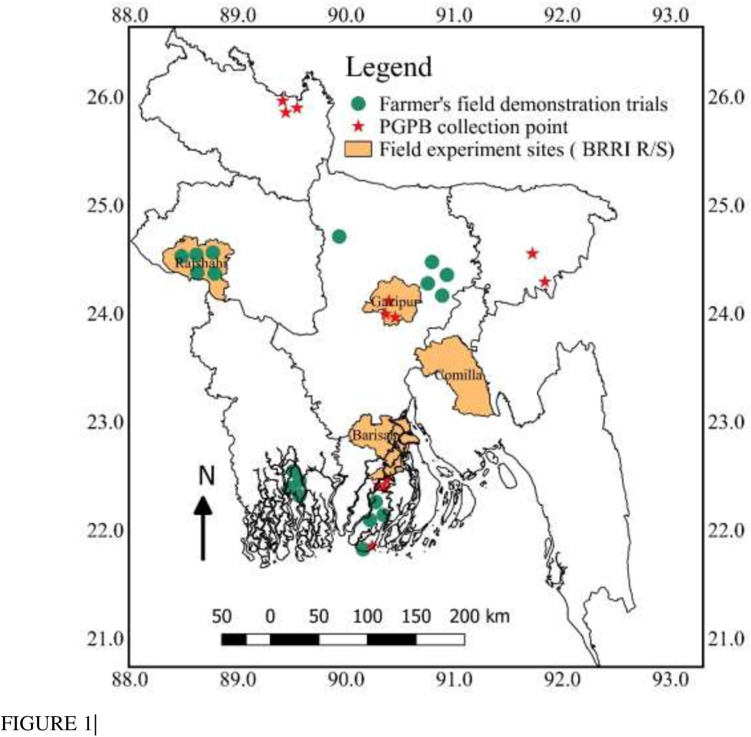
Bangladesh Map showing different locations of PGPB collections, field experiments, and farmers’ field trials.

Beneficial bacteria used in the bio-organic fertilizer (BoF) were isolated from paddy soils of Gazipur (terrace soil), Lalmonirhat (active Tista floodplain soil), and Kuakata (saline soil) (Figure 1) following protocols of Naher et al. (2009). Isolated potential bacteria were identified by 16S ribosomal RNA partial gene sequences using universal primers. Free-living N_2_ fixing bacteria were identified according to Naher et al. (2013), where forward and reverse primers were used as 8 F, 5′-AGA GTT TGA TCC TGG CTC AG-3′ and 1492R, 5′-GGT TAC CTT ACG ACT T-3′ (Versalovic et al., 1991), respectively. PSB were identified following the protocol of Panhwar et al. (2014) with forward primer F 5-AGA GTT TGA TCC TGG CTC AG-3 and reverse primer R3-ACG GCT ACC TTG TTA CGA CTT-5 (Weisburg et al., 1991). Gene sequences acquired were deposited in the sequence read archive, PRJNA662441: bio-organic fertilizer.

### Determination of N_2_ Fixation

The amount of N_2_ fixation was determined *via* NH_4_ production by the isolated strains. Identified free-living N_2_ fixing bacteria were grown in N free (NFB) broth. The composition of the NFB broth was slightly modified from Prasad et al. (2001), as (grams/liter): 5 g malic acid, 0.5-g K_2_HPO_4_, 0.2-g MgSO_4_. 7 H_2_O, 0.1-g NaCl, 0.02-g CaCl_2_, 1.64% Fe-EDTA solution (4 ml), and the pH was adjusted to 7.2. The isolated strains were cultured in the prepared broth at 30°C for 5 days on a rotary shaker (120 rpm). The bacteria culture was then centrifuged at 4000 × *g* for 5 min and filtrated through a 0.2-mm filter paper. The N was determined from the filtrate using the procedure described by Cappucino and Sherman (1992).

### Determination of P Solubilization by the Phosphate Solubilizing Bacteria

The PSB were cultured in the National Botanical Research Institute’s phosphate growth medium broth (Nautiyal, 1999) containing RP for 5 days. Exactly, 2 ml of samples was taken for P determination. The samples were first allowed to sediment for 15 min and then centrifuged at 4,000 × *g* for 5 min. The supernatant was filtered through 0.2-mm filter paper and kept at −20°C until analysis. Available P was determined using the procedure described by Murphy and Riley (1962).

### Determination of Indoleacetic Acid Production

All of the identified isolates (10 bacteria) were inoculated in nutrient broth with an addition of tryptophan (2 mg ml^–1^) and incubated at 28 ± 2°C for 3 days. The culture was centrifuged at 7,000 rpm for 7 min, and 1 ml of the supernatant was mixed with 2 ml of Salkowsky’s reagent. The IAA concentration was determined using a spectrophotometer at 535 nm.

### Evaluation of Bio-Organic Fertilizer for Plant Growth and Yield

To evaluate the performance of BoF, 16 field experiments were conducted at BRRI research stations of Gazipur, Cumilla, Barishal, and Rajshahi, and 18 farmer’s field demonstration trials were performed in different parts of Bangladesh (Figure 1) during the year 2017 to 2020. Treatments considered in the field experiments were T_1_ = control, T_2_ = full synthetic fertilizer dose (FSFD) of N, P, and K fertilizer as urea, TSP, and muriate of potash (MoP), T_3_ = BoF (applied, at 2 t ha^–1^) + 70% N as urea + 100% K as MoP, T_4_ = 70% N as urea + 100% P as TSP + 100% K as MoP, and T_5_ = BoF (2 t ha^–1^). Treatments were assigned in randomized complete block design with three replications. Farmers’ field demonstration trials were conducted in different parts of Bangladesh to check the suitability of the BoF in different types of soil (saline, floodplain, and terrace soil) and climate (Figure 1). Five field trials were conducted in drought-prone areas of Rajshahi (Poba, Durgapur, Godagari, and Shampur), eight trials in the saline soils of Dakope, Khula, and Amtoli, Barguna, four trials in flood plain soils of Kotiadi, Kishoreganj, and one trial in the terrace soil of Dhanbari, Tangail. Farmers field demonstration trials were non-replicated and treatments were as T_1_ = BoF at 2 t ha^–1^ + 70% urea N + 100% K as MoP, T_2_ = FSFD. Rice was grown in both T. Aman wet and Boro irrigated seasons. Applied fertilizer doses for FSFD were N, P, K, and S at 80–20–50 and 140–20–80 kg ha^–1^ in T. Aman wet and Boro irrigated seasons, respectively, as urea, TSP, and MoP. During the calculation of fertilizer doses, additional N and P nutrients added from BoF were deducted from respective treatments. At all of the research stations, each treatment was assigned in a 4-m × 5-m sized plot surrounded by a 50-cm bund. Approximately 30- and 45-day-old rice seedlings of high yielding modern varieties were transplanted at 20 cm × 20 cm plant spacing in the T. Aman wet and Boro irrigated seasons, respectively. Weeding and plant protection measures were done as required. Urea fertilizer was applied in three splits at equal amounts; during land preparation, maximum tillering stage, and finally at the panicle initiation stage. During final land preparation, BoF was applied along with synthetic fertilizers and incorporated with soil. The flooded water level at 5–7 cm depth was maintained during rice cultivation and then drained 21 days before rice harvesting. At the farmer’s field trials, plot size was varied from 122 to 1,336 m^2^, and high-yielding rice varieties were selected as per farmers’ choice; however, plant spacing and fertilizer application rate and method were the same. In the research stations, crops were harvested manually at maturity from a 5-m^2^ area. At farmer’s fields, crop was harvested on a whole plot basis. Grain yield was adjusted at 14% moisture content. Tiller and panicle numbers plant^–1^ were calculated from a 1-m^2^ plot area. Yield contributions of BoF were calculated from the average yield data (16 experiments) of T_3_/T_5_ treatments as

$$\%GrainyieldcontributionofBoF\left(tha^{-1}\right)$$

(1) $$=\frac{Y\,i\,e\,l\,d\,o\,f\,\left(B\,o\,F+r\,e\,d\,u\,c\,e\,d\,s\,y\,n\,t\,h\,e\,r\,t\,i\,c\,f\,e\,r\,t\,i\,l\,i\,z\,e\,r\right)}{Y\,i\,e\,l\,d\,o\,f\,B\,o\,F}\times 100$$

### Determination of Nutrient Concentration, N and P Uptake

During harvest, plant samples were separated into grain and straw followed by oven drying at 60°C for 3 days and ground before wet digestion using perchloric acid and HNO_3_ (5:2) at 100°C. Phosphorus was determined from the digested sample following the Colorimetric method (Dick and Tabatabai, 1977) and K by atomic absorption spectrometer (AA-7000, Shimadzu). Total N was determined by the Kjeldahl method (Bremner and Mulvaney, 1982). Plant nutrient uptakes were calculated in kilograms per hectare in relation to yield kilograms per hectare (Yadav et al., 2019):

$$N\,u\,t\,r\,i\,e\,n\,t\,u\,p\,t\,a\,k\,e\,N\,o\,r\,P\,o\,r\,K\,\left(k\,g\,h\,a^{-1}\right)$$

(2) $$=\frac{Nutrientcontent\left(\%\right)\times yield\left(kgha^{-1}\right)}{100}$$

### Determination of N and P Use Efficiency

Agronomic use efficiency (AE) and physiology use efficiency (PE) were calculated according to Ladha et al. (2005). PFP and RE were calculated as per Chuan et al. (2016), and nutrient HI was determined using formulae of Huang et al. (2018);

$$A\,E\,f\,o\,r\,N\,o\,r\,P\,\left(k\,g\,k\,g^{-1}\right)$$

(3) $$=\frac{G\,r\,a\,i\,n\,y\,i\,e\,l\,d\,o\,f\,n\,u\,t\,r\,i\,e\,n\,t\,a\,p\,p\,l\,i\,e\,d\,p\,l\,o\,t-G\,r\,a\,i\,n\,y\,i\,e\,l\,d\,o\,f\,c\,o\,n\,t\,r\,o\,l\,p\,l\,o\,t}{A\,m\,o\,u\,n\,t\,o\,f\,a\,p\,p\,l\,i\,e\,d\,n\,u\,t\,r\,i\,e\,n\,t}$$

$$P\,E\,N\,\left(k\,g\,k\,g^{-1}\right)$$

(4) $$=\frac{G\,r\,a\,i\,n\,y\,i\,e\,l\,d\,o\,f\,n\,u\,t\,r\,i\,e\,n\,t\,a\,p\,p\,l\,i\,e\,d\,p\,l\,o\,t-G\,r\,a\,i\,n\,y\,i\,e\,l\,d\,o\,f\,c\,o\,n\,t\,r\,o\,l\,p\,l\,o\,t}{N\,u\,p\,t\,a\,k\,e\,i\,n\,N\,a\,p\,p\,l\,i\,e\,d\,p\,l\,o\,t-N\,u\,p\,t\,a\,k\,e\,i\,n\,N\,o\,m\,i\,s\,s\,i\,o\,n\,p\,l\,o\,t}$$

$$P\,F\,P\,f\,o\,r\,N\,o\,r\,P\,\left(k\,g\,k\,g^{-1}\right)$$

(5) $$=\frac{G\,r\,a\,i\,n\,y\,i\,e\,l\,d\,o\,f\,n\,u\,t\,r\,i\,e\,n\,t\,a\,p\,p\,l\,i\,e\,d\,p\,l\,o\,t}{A\,m\,o\,u\,n\,t\,o\,f\,a\,p\,p\,l\,i\,e\,d\,n\,u\,t\,r\,i\,e\,n\,t}$$

$$R\,E\,f\,o\,r\,N\,o\,r\,P\,\left(k\,g\,k\,g^{-1}\right)$$

(6) $$=\frac{N\,u\,t\,r\,i\,e\,n\,t\,u\,p\,t\,a\,k\,e\,\left[c\,p\,s\,b\,r\,e\,a\,k\right]\,f\,e\,r\,t\,i\,l\,i\,z\,e\,d\,p\,l\,o\,t-N\,u\,t\,r\,i\,e\,n\,t\,u\,p\,t\,a\,k\,e\,c\,o\,n\,t\,r\,o\,l\,p\,l\,o\,t}{A\,m\,o\,u\,n\,t\,o\,f\,a\,p\,p\,l\,i\,e\,d\,n\,u\,t\,r\,i\,e\,n\,t}$$

$$H\,I\,f\,o\,r\,N\,o\,r\,P\,\left(k\,g\,k\,g^{-1}\right)$$

(7) $$=\frac{N\,u\,t\,r\,i\,e\,n\,t\,a\,c\,c\,u\,m\,u\,l\,a\,t\,i\,o\,n\,i\,n\,g\,r\,a\,i\,n}{A\,m\,o\,u\,n\,t\,o\,f\,n\,u\,t\,r\,i\,e\,n\,t\,u\,p\,t\,a\,k\,e\,\left(G\,r\,a\,i\,n+S\,t\,r\,a\,w\right)}\times 100$$

### Determination of Soil Nutrient Concentration

After completion of eight crop cycles, soil (0–20 cm depth) was collected from the experimental field of BRRI Gazipur and BRRI regional station Cumilla for determination of organic carbon (OC), total N, available P, exchangeable K, and a total population of PSB and free-living N_2_ fixing bacteria. Before chemical analyses, soil was air-dried, ground, and sieved (2 mm). Organic C was determined by following the method of Walkley and Black (1934). Total N was determined by the Kjeldahl digestion method (Bremner and Mulvaney, 1982). Available P was determined by the extraction method of Bray and Kurtz (1945) following colorimetric in a spectrophotometer. Exchangeable K was extracted using ammonium acetate buffer at pH of 7 (Page et al., 1982) and determined by AAS (AA-7000, Shimadzu). The population of free-living N_2_ fixing bacteria and PSB was determined by serial dilution of soil sample and spread plate technique in specific media plates described by Naher et al. (2009) and Panhwar et al. (2014), respectively.

### Statistical Analyses

Biochemical analyses in the laboratory were arranged in a completely randomized design with five replicates. The quantitative results of the laboratory and field experiments were subjected to an analysis of variance, and means of different treatments were compared at a 5% level of significance by Duncan’s Multiple Range Test using STAR 2.01 (Statistical Tool for Agricultural Research [STAR], 2014) statistical program. A combined statistical analysis tool (*t*-test) was applied to compare the data generated in the farmers’ field demonstration trials.

## Results

### Potentiality of the PGPB to Supplement Reactive N and Solubilize Rock Phosphate

Bio-organic fertilizer prepared using a consortium of 10 microaerophilic PGPB isolated from the floodplain, terrace, and saline rice soil (Figure 1) was subjected to 16S ribosomal RNA partial gene sequences and identified commonly as *Bacillus* and *Paenibacillus* spp. Most PGPB were proficient in N_2_ fixing, P solubilizing, and produced IAA (Table 1). The biological N_2_ fixing capability of the isolated strains ranged from 0.07 to 0.14%. Among the isolates, *Proteus* sp. and *Paenibacillus polymyxa* fixed the highest amounts of atmospheric N_2_. However, results were statistically similar with *B. subtilis*, *B. cereus*, *Paenibacillus* sp., and other two *Bacillus* spp. The ability of PSB for phosphate solubilization from RP ranged from 0.01 to 0.12%. Potential P solubilizers were *B. pumilus* (0.08%), *B. cereus* (0.11%), *Paenibacillus* sp. (0.11%), and one *Bacillus* sp. (0.12%). The highest amount of IAA (0.36%) was produced by both *P. polymyxa* and *B. cereus*; nevertheless, all the identified strains had the potential to produce this particular plant growth hormone.

**TABLE 1:** Identification and biochemical properties of bacterial strains used in bio-organic fertilizer.

**Bacteria strains**	*ID: SRA/EMBL-NCBI	**Similarity(%)	**Biochemical properties (g kg^–1^)**		
			***IAA	**Total N**	**Extractable P**
					
*Paenibacillus* sp.	SRX9106118	97	0.3 b	0.7 ab	1.1 a
*Paenibacillus polymyxa*	SRX9106116	98	4.0 a	1.4 a	0.5 ab
*Bacillus* sp.	SRX9106117	85	0.3 b	1.0 a	1.2 a
*Bacillus mycoides*	SRX9106115	97	0.1 b	0.7 ab	0.4 b
*Proteus* sp.	SRX9106121	95	0.1 b	1.3 a	0.1 b
*Bacillus cereus*	SRX9106122	95	3.6 a	0.8 ab	1.1 a
*Bacillus subtilis*	JQ820255	97	0.1 b	0.8 ab	0.4 b
*Bacillus pumilus*	SRX9106119	97	0.3 b	0.4 b	0.8 ab
*Bacillus* sp.	SRX9106120	85	0.2 b	1.2 a	0.7 ab
*Bacillus* sp.	SRX9106114	85	0.1 b	1.1 a	0.4 b

Different lowercase letters within the column denote significant differences between the treatments according to ANOVA and Duncan’s multiple range test at a 5% level of significance. *Sequence Read Archive/European Molecular Biology Laboratory databank— National Center for Biotechnology Information. **Based on nucleotide sequence database (National Center for Biotechnology Information). ***Indoleacetic acid.

### Grain Yield Response to the Applied Bio-Organic and Synthetic Fertilizer

**FIGURE 2:**
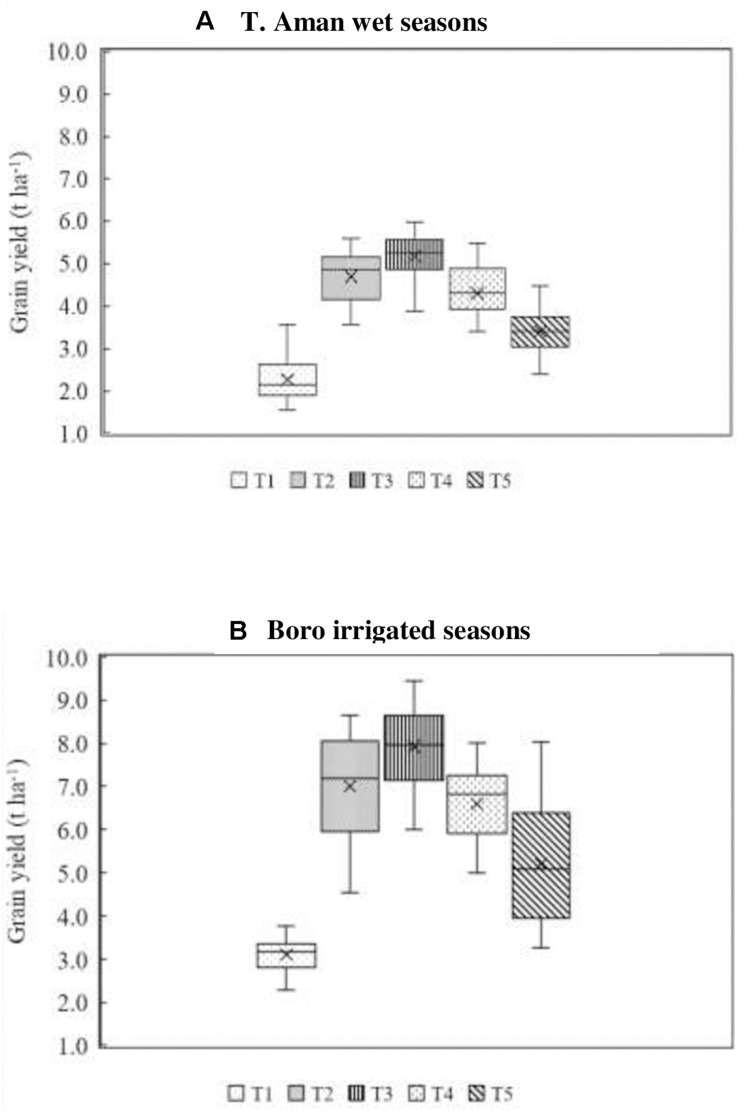
Effect of fertilizer management practices on grain yield of rice. Grain yield data obtained from field experiments conducted at BRRI research stations (eight experiments in each season) during years 2017–2020. **(A)** T. Aman wet seasons, **(B)** Boro irrigated seasons. Line that divides box represents median of data. End of box shows upper and lower quartiles (*n* = 24 for each season). T_1_ = control, T_2_ = FSFD: full synthetic fertilizer dose of N, P, and K at 80–20–50 and 140–20–80 kg ha^–1^ as urea, triple super phosphate (TSP), and muriate of potash (MoP) in T. Aman wet and Boro irrigated seasons, respectively; T_3_ = BoF (2 t ha^–1^) + 70% N as urea + 100% K as MoP, T_4_ = 70% N as urea + 100% P as TSP + 100% K as MoP; T_5_ = sole BoF at 2 t ha^–1^. BoF was added as dry weight basis.

Field experiments (18) were conducted at different research stations of BRRI in both T. Aman wet and Boro irrigated seasons. Average grain yield data obtained from different replicated treatments of all experiment sites (eight at each season) were arranged in a box plot. Figure 2A illustrates the results of T. Aman wet seasons where an average 10.44% higher grain yield was obtained in the BoF (2 t ha^–1^) + 70% N as urea + 100% K as MoP treatment (T_3_) compared with full synthetic fertilizer (T_2_) treatment. Grain yield varied from 3.89 to 5.98 t ha^–1^ with an average value of 5.18 in T_3_ treatment. Conversely, in the full synthetic N, P, and K fertilizer treatment (T_2_), grain yield ranged from 3.56 to 5.60 t ha^–1^ with an average of 4.69 t ha^–1^. Reduction of 30% N from the full dose of urea fertilizer (T_2_) reduced 8% of average grain yield in T_4_ treatment. Nonetheless, BoF treatment (T_5_) alone was not capable of producing grain yield comparable with FSFD treatment (T_2_), although the total contribution of bio-organic fertilizer on grain yield was 65.83% in T. Aman wet seasons. The lowest average grain yield obtained in the control plot was 2.2 t ha^–1^.

In general, grain yield was higher in the Boro irrigated compared with T. Aman wet seasons (Figure 2B). The yield trend of Boro irrigated seasons (average eight experiments) followed a similar pattern of T. Aman wet seasons, where grain yield in BoF + 70% N as urea + 100% K as MoP (T_3_) ranged from 6.0 to 8.9 t ha^–1^ with an average of 7.87 t ha^–1^. In this treatment (T_3_), 25% grain yield value below the median was 7.22 t ha^–1^, and above the 75th percentile value was 8.64 t ha^–1^. On average, 13% higher grain yield was obtained in the T_3_ treatment compared with the FSFD application. In the FSFD treatment (T_2_), grain yield varied from 4.54 to 8.66 t ha^–1^, and the average yield was 6.97 t ha^–1^. In the Boro irrigated seasons, a reduction of 30% N from FSFD treatment (T_2_) reduced 19% grain yield in T_4_. Nevertheless, the application of BoF alone gave an average 5.21 t ha^–1^ grain yield. The total contribution of bio-organic fertilizer on grain yield was 66.79% in Boro irrigated seasons. The lowest average grain yield was 3.11 t ha^–1^ recorded in the control treatment.

### Effect of Fertilizer Management Practices on Plant Nutrient Uptake

Bio-organic and synthetic fertilizer management practices have significant effects on plant N, P, and K uptakes. Across all field experiments and irrespective of seasons, the highest average plant N uptake was recorded in both the BoF (2 t ha^–1^) + 70% urea N + 100% MoP (T_3_) and a full dose of synthetic fertilizers (T_2_) followed by sole BoF (T_5_) application. In T_4_, a reduction of 30% N from FSFD (T_2_) significantly reduced N uptake, and the lowest N uptake was recorded in the fertilizer control treatment (T_1_). Table 2 demonstrates the nutrient uptake in T. Aman wet seasons, where N uptake in T_3_ treatment ranged from 64 to 110 kg ha^–1^ with an average value of 92 kg ha^–1^. Correspondingly, in the FSFD (T_2_) practice, the average N uptake was 90 kg ha^–1^, and it ranged from 63 to 106 kg ha^–1^. Reduction of 30% N from the full dose of synthetic fertilizer (T_2_) reduced 30.41% average N uptake in T_4_. Nevertheless, 42–100 kg ha^–1^ N uptake was recorded in the sole BoF use.

**TABLE 2:** Contribution of bio-organic and synthetic N, P, and K fertilizers on nutrient uptake (kilograms per hectare) in T. Aman wet seasons and Boro irrigated seasons.

**Treatment**	**T. Aman wet seasons**			**Boro irrigated seasons**		

	**Uptake (kg ha^–1^)**					
	**N**	**P**	**K**	**N**	**P**	**K**
T_1_	29 d	9 c	30 c	4 d	2 d	6 d
T_2_	90 a	19 b	67 a	12 b	4 b	10 b
T_3_	92 a	21 a	69 a	13 a	4 a	11 a
T_4_	69 c	17 b	54 b	7 c	3 c	8 bc
T_5_	79 b	19 b	55 b	17 c	3 c	14 c
CV (%)	14.4	16.4	14.5	16.6	17.5	18.5
*P*-value	0.00	0.00	0.00	0.00	0.00	0.00

*Data generated from field experiments conducted at BRRI research stations during years 2017–2020. Different lowercase letters within the column denote significant differences between the treatments according to ANOVA and Duncan’s multiple range test at a 1% level of significance. T_1_ = Fertilizer control, T_2_ = full synthetic fertilizer dose of N, P, and K at 80–20–50 as urea, triple superphosphate (TSP), and muriate of potash (MoP); T_3_ = BoF (2 t ha^–1^) + 70% N as urea + 100% K as MoP, T_4_ = 70% N as urea + 100% P as TSP + 100% K as MoP T_5_ = Sole BoF at 2 t ha^–1^ BoF was added as dry weight basis.*

Variation in P uptake was found among the treatments. A higher range of P uptake (13–26 kg ha^–1^) with an average value of 21 kg P ha^–1^ was obtained in T_3_, where RP was the source of P nutrient. However, P uptake ranged from 10 to 24 kg ha^–1^ in synthetic fertilizer (TSP) applied treatment (T_2_). Conversely, in the sole BoF treatment (T_5_), P uptake was 12 to 24 kg ha^–1^, and average P uptake was 19 kg ha^–1^, which proved a copious supply of available P from RP during the plant growth period. Average K uptake was high in both FSFD and BoF (2 t ha^–1^) + 70% urea N + 100% K as MoP (T_3_) compared with other fertilizer practices. Reduction of 30% N from FSFD reduced average 12% P and 24% K uptake in the T. Aman wet seasons. The lowest N, P, and K uptakes were found in the control treatment (Table 2).

In the Boro irrigated seasons, rice grain yield and plant nutrient uptakes were found higher than T. Aman wet seasons. Nutrient uptakes by rice plant in Boro irrigated seasons were illustrated in Table 2. Average N uptakes were 142 and 131 kg ha^–1^ in the T_3_ and T_2_ practices, respectively. N uptakes varied from 96 to 192 and 92 to 188 kg ha^–1^ in T_3_ and T_2_ treatment, respectively, whereas they ranged from 88 to 141 and 71 to 139 kg ha^–1^ in the T_4_ (30% reduced N from FSFD) and T_5_ (BoF) treatments, respectively. Reduction of 30% N from T_2_ reduced 15% N uptake in T_4_, whereas reduction of 70% synthetic N in the BoF (T_5_) in comparison with T_3_ reduced only 31% N uptake in Boro irrigated seasons, which proved Nr increment *via* bio-organic fertilizer. The average high P uptake was 35 kg ha^–1^, and it ranged from 22 to 44 kg ha^–1^ in the T_3_, although hardly soluble RP was used as the source of P. In contrast, the average P uptake was 29 kg ha^–1^ and ranged from 20 to 40 kg ha^–1^ in the TSP applied FSFD treatment (T_2_). In general, N, P, and K uptakes were highest in the T_3_ followed by T_2_ practice. Reduction of 30% N from FSFD reduced 8.3% N, 26% P, and 10.38% K uptakes in T_4_ treatment during the Boro irrigated seasons.

### Effect of Fertilizer Management Practices on Agronomic N Use Efficiency, Physiological N Use Efficiency, N Recovery Efficiency, Partial Factor Productivity of N, and N Harvest Index

**FIGURE 3:**
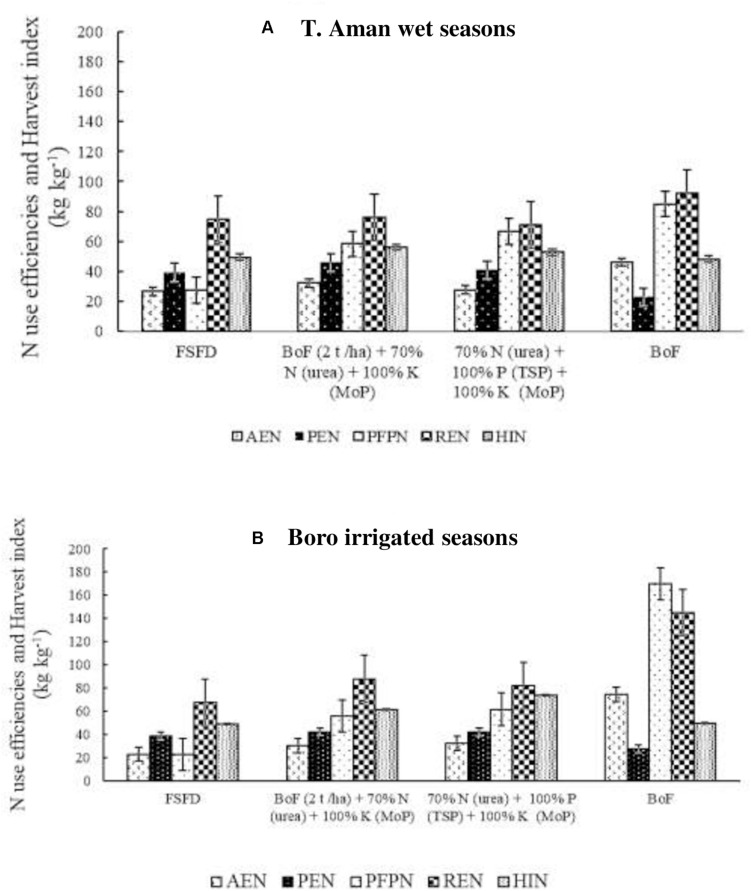
Effect of fertilizer management practices on agronomic efficiency of N (AE_N_), physiological efficiency of N (PE_N_), partial factor productivity of N (PFP_N_), recovery efficiency of N (RE_N_), and N harvest index (HI_N_). Data generated from field experiments conducted at BRRI research stations (eight experiments in each season) during years 2017–2020. **(A)** T. Aman wet seasons, **(B)** Boro irrigated seasons. Error bar indicates standard error (*n* = 24). FSFD, full synthetic fertilizer dose of N, P, and K at 80–20–50 and 140–20–80 kg ha^–1^ as urea, triple superphosphate (TSP), and muriate of potash (MoP) in T. Aman wet and Boro irrigated seasons, respectively. BoF (2 t ha^–1^) + 70% N as urea + 100% K as MoP; 70% N as urea + 100% P as TSP + 100% K as MoP. Sole BoF at 2 t ha^–1^. BoF was added as dry weight basis.

Across the eight field experiments in T. Aman wet seasons, the average agronomic N use efficiency (AE_N_) was higher in the bio-organic fertilizer applied plots. The highest average AE_N_ (46 kg kg^–1^) was recorded in the sole BoF (T_5_) followed by the T_3_ (32 kg kg^–1^) treatment (Figure 3A). Comparable N use efficiency was found in T_2_ (27 kg kg^–1^) and T_4_ treatment. However, 30% synthetic N reduction in T_3_ increased by 22% AE_N_ over T_2_ treatment that evidenced BNF contribution. Physiological N use efficiency (PE_N_) was also higher in T_3_ (46 kg kg^–1^) compared with T_2_ (39 kg kg^–1^), and the lowest PE_N_ was found in T_5_ treatment. The average partial factor productivity of N (PFP_N_) was high in T_3_ (85 kg kg^–1^) followed by T_4_. The lowest PFP_N_ (27 kg kg^–1^) was found in the T_2_ treatment. The highest average N recovery efficiency (RE_N_) (56 kg kg^–1^) was recorded in the BoF (T_5_) treatment, whereas, in the other three treatments, RE_N_ was almost identical. In N harvest index (HI_N_), comparable values were obtained among the treatments (Figure 3A).

In the Boro irrigated seasons (Figure 3B), among the eight field experiments, a high average AE_N_ (75 kg kg^–1^) was recorded in the BoF treatment (T_5_). AE_N_ increased by approximately 30% in T_3_ parallel to FSFD (T_2_) treatment. FSFD treatment exhibited the lowest average AE_N_ (23 kg kg^–1^) in the Boro irrigated seasons. A similar PE_N_ value (42 kg kg^–1^) was obtained in both T_3_ and T_4_ treatments, and the lowest value was found in the T_5_ (28 kg kg^–1^) treatment. Application of full synthetic fertilizer (T_2_) showed 8% less PE_N_ compared with T_2_ and T_3_ treatments. The average partial factor productivity of applied N was highest (170 kg kg^–1^) in the BoF (T_5_), followed by T_4_ (62 kg kg^–1^) and T_3_ (56 kg ka^–1^) treatments. A full dose of synthetic fertilizer application (T_2_) had the lowest (23 kg kg^–1^) PFP_N_. RE_N_ was almost comparable in both FSFD (T_2_) and BoF (2 t ha^–1^) + 70% N as urea + 100% K as MoP treatment (T_3_); however, an increment was observed in the T_3_ treatment. The highest RE_N_ (145 kg kg^–1^) was obtained in the BoF (T_5_) treatment. Compared with T. Aman wet seasons, HI_N_ was higher in the Boro irrigated seasons, and the highest HI_N_ (74 kg kg^–1^) was obtained in the T_4_, trailed by T_3_ (62 kg kg^–1^) treatment. Comparable values of HI_N_ (49 kg kg^–1^) were obtained due to the application of full synthetic fertilizer (T_1_) or sole bio-organic fertilizer (T_5_) among the field experiments (Figure 3B).

**FIGURE 4:**
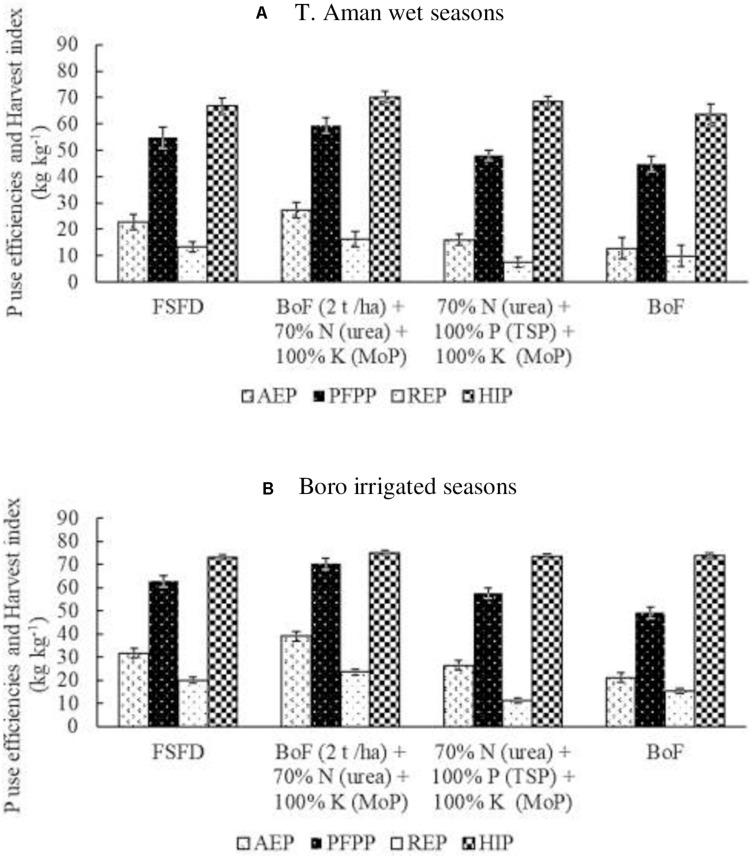
Effect of fertilizer management practices on agronomy use efficiency of P (AE_P_), partial factor productivity of P (PFP_P_), recovery efficiency of P (RE_P_), and P harvest index (HI_P_). Data generated from field experiments conducted at BRRI research stations (eight experiments in each season) during years 2017–2020. **(A)** T. Aman wet seasons, **(B)** Boro irrigated seasons. Error bar indicates standard error (*n* = 24). FSFD, full synthetic fertilizer dose of N, P, and K at 80–20–50 and 140–20–80 kg ha^–1^ as urea, triple superphosphate (TSP), and muriate of potash (MoP) in T. Aman wet and Boro irrigated seasons, respectively. BoF (2 t ha^–1^) + 70% N as urea + 100% K as MoP; 70% N as urea + 100% P as TSP + 100% K as MoP. Sole BoF at 2 t ha^–1^. BoF was added as dry weight basis.

Triple superphosphate and RP were used as the source of P nutrient in this study. Irrespective of the source, equal amounts of P were added to all treatments. Figure 4A explains the average data generated in T. Aman wet seasons from the eight field experiments and found that agronomic use efficiency of P was highest (27 kg kg^–1^) in the T_3_ treatment, where RP was used as P source during the formulation of bio-organic fertilizer. Nonetheless, 23 and 16 kg kg^–1^ agronomic P use efficiency (AE_P_) were recorded in the T_2_ and T_4_ treatments, respectively, where TSP was the source of P. Approximately 12 kg kg^–1^ AE_P_ was found due to the application of sole BoF (T_5_). Similar to the trend of AE_P_, the average productivity of P fertilizer [partial factor productivity of P (PFP_P_)] was 7% higher in the T_3_ (59 kg kg^–1^) followed by T_2_ (55 kg kg^–1^) treatment. Comparable PFP_P_ was recorded in the T_4_ (48 kg kg^–1^) and T_5_ (45 kg kg^–1^) treatments. RE_P_ was also higher in the T_3_ (16 kg kg^–1^) where the P source was RP. Nevertheless, 13 kg kg^–1^ RE_P_ was found in the T_2_, 9.91 kg kg^–1^ in the T_5_, and the lowest RE_P_ (7 kg kg^–1^) was exhibited in the T_4_ treatment. In both circumstances, P source was TSP. A similar HI_P_ (67 kg kg^–1^) was found in the TSP applied treatments (T_2_ and T_4_), although a little higher (4%) average HI_P_ (70 kg kg^–1^) was obtained in the RP added BoF (2 t ha^–1^) + 70% N as urea + 100% K as MoP treatment (T_3_). In the BoF (T_5_) treatment, HI_P_ was 64 kg kg^–1^.

In the Boro irrigated seasons, average P nutrient use efficiencies and HI_P_ were higher than in the T. Aman wet seasons (Figure 4B). The highest AE_P_ of 39 kg kg^–1^ P was found in the T_3_ treatment, where the P source was RP from bio-organic fertilizer. Full synthetic fertilizer treatment (FSFD) exhibited an AE_P_ of 32 kg kg^–1^, whereas T_4_ treatment gave 27 kg kg^–1^ AE_P_. Approximately 22% AE_P_ increased in T_3_ compared with T_2_ treatment, and a reduction of 30% N from FSFD reduced 23% AE_P_ in the T_4_ treatment. BoF (T_5_) exhibited the lowest average AE_P_ (21 kg kg^–1^) in the Boro irrigated seasons. Partial factor productivity of P fertilizer was also higher (70 kg kg^–1^) in the BoF (2 t ha^–1^) + 70% N as urea + 100% K as MoP (T_3_), where P source was RP. Synthetic fertilizer (TSP) application in T_2_ and T_4_ treatments gave an average of 63 and 58 kg kg^–1^ PFP_P_, respectively. Sole application of BoF had 49 kg kg^–1^ PFP_P_. RE_P_ was 24 kg kg^–1^ in T_3_ (P source was RP) and 20 kg kg^–1^ in T_2_ (P source was TSP). Conversely, a 30% reduction of synthetic N fertilizer reduced 81% RE_P_ in T_4_ compared with FSFD treatment (T_2_). The lowest RE of 11 kg kg^–1^ was found in the T_4_ treatment. In the Boro irrigated seasons, the average HI_P_ was analogous regardless of treatments.

### Farmers’ Field Demonstration Trials

Partial factor productivity of N, P fertilizer, plant growth, and yield parameters were subjected to pooled analysis *via* a two-tailed *t*-test with the following hypothesis: *H*_0_: BoF (2 t ha^–1^) + 70% N as urea + 100% K as MoP treatment (T_1_) supplemented by 30% Nr, which had eliminated 100% TSP in rice production and produced higher plant height, tiller number, panicle number, grain yield, and PFP_N_ and PFP_P_ compared with full-dose synthetic fertilizer (FSFD) application (T_2_), *H*_a_: BoF (2 t ha^–1^) + 70% N as urea + 100% K, (T_1_), which was not able to supplement 30% Nr, had eliminated 100% TSP in rice production and produced lower plant height, tiller number, panicle number, grain yield, and PFP_N_ and PFP_P_ compared with FSFD treatment (T_2_). The statistical analyses obtained from 18 field demonstration trials are illustrated in Table 3. Irrespective of soil and climate across the farmers’ field trials, T_1_ produced higher average plant height (5.4%), tiller number (6.1%), panicle number (8.8%), and grain yield (7.2%) compared with the full dose of synthetic fertilizer (T_2_) application. Partial factor productivity of N and P were calculated, and it was found that BoF (2 t ha^–1^) + 70% N as urea + 100% K treatment gave 8.03 and 6.4% higher PFP_N_ and PFP_P_, respectively, as compared with FSFD practice. The results mentioned earlier rejected the alternate hypothesis at a 0.05% level of significance and provided evidence of the efficacy of bio-organic fertilizer that supplemented 30% Nr and 100% TSP in rice production over a full dose of chemical fertilizer treatment.

**TABLE 3:** Plant height, tiller number, panicle number, grain yield, and partial factor productivity of N and P as influenced by bio-organic and synthetic fertilizers at farmers’ field demonstration trials (18) during years 2017–2020 (*n* = 18).

**Parameters**	**Plant height**		**Tiller number/m^2^**		**Panicle number/m^2^**		**Grain yield (t ha^–1^)**		**PFP_N_**		**PFP_P_**	
	**T_1_**	**T_2_**	**T_1_**	**T_2_**	**T_1_**	**T_2_**	**T_1_**	**T_2_**	**T_1_**	**T_2_**	**T_1_**	**T_2_**
						
Mean	89.35	84.8	245	230	222	204	5	4.66	58.10	53.78	50	47
Variance	23.39	36.58	5603	5329	645	5986	1.968	1.958	273	295	196	196
T stat	2.098		2.649		2.51		2.874		3.030		1.729	
P (T <= t) two-tail	0.049		0.015		0.021		0.0097		0.0068		0.011	
T critical two-tail	2.093		2.093		2.093		2.093		2.093		2.093	

*T_1_, BoF (2 t ha^–1^) + 70% N as urea + 100% K as MoP, T_2_, FSFD (full synthetic fertilizer dose of N, P, and K fertilizer as urea, triple superphosphate, and muriate of potash. N, P, K, and S at 80–20–50 and 140–20–80 kg ha^–1^ applied in the T. Aman wet and Boro irrigated seasons, separately). BoF, bio-organic fertilizer applied as dry weight basis*.

### Effect of Bio-Organic and Synthetic Fertilizer on Soil Biochemical Properties

Different fertilizer management practices (4 years) affected soil biochemical properties (Table 4). Bio-organic fertilizer was an organic-based component, and it contained 15% biochar as an ingredient, which improved soil carbon from 6 to 13% over synthetic fertilizer. Application of synthetic fertilizer N, P, or K or bio-organic fertilizer improved soil N, P, K, and S concentration compared with control treatment. The population of free-living N_2_ fixing bacteria and PSB significantly increased over synthetic fertilizer treatment, which proved strain survivability in the vicinity. In general, the application of bio-organic fertilizer enriched soil carbon and biology with beneficial bacteria.

**TABLE 4:** Effect of bio-organic and synthetic N, P, K fertilizers on soil biochemical properties.

**Treatments**	**N (g kg^–1^)**	**OC (g kg^–1^)**	**Extractable. P (mg kg^–1^)**	**Exch. K (mg kg^–1^)**	**Avail. S (mg kg^–1^)**	**Free-living N_2_ fixing bacteria (CFU g^–1^ soil)**	*PSB (Cfu g^–1^ soil)
T_1_	1.1 ± 0.1	15 ± 1	22 ± 3.2	42.9 ± 3.9	21 ± 0.3	2.4 × 10^4^	6.2 × 10^4^
T_2_	1.4 ± 0.1	15 ± 3	30 ± 3.6	58.5 ± 4.2	23 ± 1.0	4.1 × 10^5^	8.1 × 10^5^
T_3_	1.4 ± 0.08	17 ± 1	31 ± 0.3	58.5 ± 2.3	24 ± 0.4	5.3 × 10^8^	2.8 × 10^8^
T_4_	1.3 ± 0.1	15 ± 2	26 ± 1.2	50.7 ± 4.0	22 ± 1.0	2.2 × 10^4^	3.4 × 10^5^
T_5_	1.3 ± 0.09	16 ± 1	31 ± 2.2	58.5 ± 3.9	24 ± 1.5	6.1 × 10^7^	2.9 × 10^8^

Average values generated from the field experiments conducted at BRRI research station Gazipur and Cumilla during years 2017–2020 (two cycles each year). (Standard deviation, n = 24) T_1_, BoF (2 t ha^–1^) + 70% N as urea + 100% K as MoP, T_2_, FSFD (full synthetic fertilizer dose of N, P, and K fertilizer as urea, triple superphosphate, and muriate of potash. N, P, K, and S at 80–20–50 and 140–20–80 kg ha^–1^ applied in the T. Aman wet and Boro irrigated seasons, separately). BoF, bio-organic fertilizer applied as dry weight basis. *PSB, phosphate solubilizing bacteria.

## Discussion

Results of our field experiments and farmers’ field trials proved that PGPB could compensate at least 30% Nr and eliminate 100% TSP requirement of rice plants without sacrificing yield. In the present study, potential indigenous PGPB were isolated from the floodplain, terrace, and saline soils and identified mostly as *Bacillus* and *Paenibacillus* spp. The ability of BNF P solubilization and IAA production by these potential bacteria were quantified and found that all the strains have those relevant mechanisms to complement plant nutrients and augment plant growth. Our findings were concomitant with the research results of Kuan et al. (2016), Weselowski et al. (2016), Xie et al. (2016), and Li et al. (2017) where elucidated *Bacillus* and *Paenibacillus* spp. mediated crop growth promotion with the involvement of those single or combined mechanisms. Nevertheless, PGPB-mediated nutrient acquisition and mechanisms of crop growth promotion in food crops were also documented by Brown (1974), Burr et al. (1978), Teintze et al. (1981), Lin et al. (1983), and Jacoby et al. (2017).

In the formulated bio-organic fertilizer, the BNF capacity was varied among the PGPB isolates, and our biochemical analysis proved *P. polymyxa*, *Proteus* sp., and *Bacillus* spp. have a higher capacity of NH_4_ production. The prospect of *Paenibacillus* sp. to be used as bioinoculant was reported by Goswami et al. (2015), where the strain produced 3.6 mg kg^–1^ NH_4_ within 96 h, and the rate of NH_4_ production was in agreement with our study. Individual N_2_ supplying capability of *P. polymyxa*, *B. cereus*, and *Proteus* sp. in association with cereal crops were also reported by Jimtha et al. (2017) and Akintokun et al. (2019). The potentiality of N_2_ fixation by applied inoculant was reflected in the plant N uptake. The study report showed that regardless of seasons, N uptake was similar in both full synthetic fertilizer (T_2_) and bio-organic fertilizer (2 t ha^–1^) + 70% N as urea + 100% K as muriate of potash applied treatment (T_3_). Although 70% synthetic N was reduced in the bio-organic fertilizer treatment (T_5_) in comparison to T_3_ treatment, N uptake reduction was only 16–31% in T_5,_ which firmly supported the incidence of adequate Nr in bio-organic fertilizer and also established the N delivering capacity of *Bacillus* and *Paenibacillus* spp. throughout the plant growing seasons. Kuan et al. (2016) also reported the BNF potentiality of several *Bacillus* spp. and found *B. pumilus* alone supplemented 30.5% N requirement in maize. Furthermore, AE_N_, PE_N_, RE_N_, and HI_N_ were higher in the bio-organic fertilizer applied treatments. Incidence of 30% synthetic N reduction increased 21% RE in T_4_ compared with full synthetic fertilizer (T_2_) treatment, and the same reduction of synthetic N had increased 29% RE in the bio-organic with reduced N and TSP omitted (T_3_) treatment and repeatedly proved the efficacy of applied free-living N_2_ fixing bacteria to supplement Nr fertilizer. In comparison with synthetic and bio-organic fertilizer, PFP_N_ of bio-organic fertilizer was higher. Besides research results, a significantly (*p* < 0.05) higher N partial factor productivity value of bio-organic fertilizer (2 t ha^–1^) + 70% N as urea + 100% K as muriate of potash was obtained in the farmers’ field demonstration trials that again supported the efficacy of bio-organic fertilizer to complement synthetic N. According to Dobermann (2007) and Fixen (2007), the best nutrient management practice and agronomic use efficiency of N synthetic fertilizer should be greater than 25 kg kg^–1^, and RE should be 50–80%. Results of our field experiments provide evidence that the treatment comprised bio-organic fertilizer crossed the ranges described earlier. Furthermore, the values of N use efficiencies and partial factor productivity consequent in our study were in the array described by Che et al. (2015).

The TSP is a commonly used synthetic fertilizer utilized to accomplish P nutrition in rice production. RP is the natural source of P, and it is the main ingredient of TSP fertilizer; however, the hardly soluble criteria of RP limited its use in rice production. In the study, TSP fertilizer was substituted by RP with the assistance of *B. pumilus, B. cereus*, and *Paenibacillus* sp., and these PGPB strains effectively abounded an adequate amount of bioavailable P from RP during the rice-growing period. Microbial-mediated P mobilization was mentioned by many scientists (Nahas, 1996; Rodrıìguez and Fraga, 1999; Plassard et al., 2011; Panhwar et al., 2013; Ahemad and Kibret, 2014). In the study, we noticed *Paenibacillus* and *Bacillus* spp. solubilized 0.1–1.2 g kg^–1^ P from RP. Several studies supported our findings, where they reported RP solubilizing capacity of *B. pumilus* was 0.35 g kg^–1^ (Dipta et al., 2017) and *B. cereus* 0.2 g kg^–1^ (Akintokun et al., 2019). However, Din et al. (2020) reported *P. polymyxa* to solubilize 2.6 g kg^–1^ P from tricalcium phosphate. Furthermore, P solubilization by *P. polymyxa* was strongly supported by Xie et al. (2016); Hao and Chen (2017), and Hashem et al. (2019). Similar to the N nutrient, P uptake and use efficiencies were apparent in bioavailable P from RP during plant growth. In our study, approximately 21% lower P uptake was noticed in T_2_ compared with T_3_ treatment that revealed spontaneously bioavailable P in the RP added treatment. We also found that, regardless of P sources, comparable values of AE_P_ and RE_P_ were obtained in both TSP fertilizer and RP applied treatments. However, lower AE_P_ and PFP_P_ values were obtained in the sole bio-organic fertilizer treatment (T_5_) due to low grain yield in the N and K deficient conditions.

The values obtained for RE_P_ were concomitant with Baligar et al. (2001), Pheav et al. (2003), and Roberts and Johnston (2015). A parallel HI_P_ also proved the similar trend of P nutrient from both sources. Partial factor productivity of fertilizer was used to evaluate the fertilizer use efficiency (Chuan et al., 2016), and the partial factor productivity of P obtained from both farmers’ field trials and field experiments (total 36) proved the efficacy of applied bio-organic fertilizer to replace 100% TSP in rice production.

Besides PGPB-mediated BNF and P solubilization from RP, the contribution of IAA was remarkable for plant growth promotion (Shen et al., 2016). A considerable amount of IAA production by *B. cereus* (Akintokun et al., 2019) and *P. polymyxa* (Liu et al., 2019) were concomitant with our research result. This particular growth hormone modulated rice root architecture (Biswas et al., 2000) that enables higher nutrient acquisition for plants and impacts crop growth and yield (Di Benedetto et al., 2017). Significantly taller plants were noticed in the bio-organic treated farmers’ field demonstration trials, which might be the resultant effect of IAA production by the *Bacillus* and *Paenibacillus* spp. Across the 18 field experiments, treatment contained bio-organic fertilizer (2 t ha^–1^) with 30% reduced N, 100% K, and TSP eliminated treatment (T_3_) produced 10–13% higher grain yield compared with full synthetic fertilizer treatment. Grain yield increment aligned with the result of Schütz et al. (2018), which reported approximately 14% of crop yield response due to microbial inoculant application from the meta-analysis of 66 experiments in the tropical climate. Depending on seasons, 30% Nr cutoff from full synthetic fertilizer treatment (T_2_) reduced approximately 8–19% grain yield in the synthetic fertilizer treatment (T_4_); nevertheless, the same reduction of Nr in bio-organic fertilizer treatment (T_3_) evidenced 13% higher grain yield. Yield increment incident has proven the spontaneous Nr and P supply by PGPB, and there was no hidden hunger for these nutrients. Field experiments result showed that the total contributions of bio-organic fertilizer on grain yield production were 65.83% in T. Aman wet seasons and 66.79% in the Boro irrigated seasons. Recently, rice yield increases due to integrated approaches of organic, synthetic, and microbial inoculants, which have been reported by Yadav et al. (2019). A combination of both functional traits was more effective than single traits. Combined activity of nutrient supplement and plant growth promotion by PGPB were reflected on plant nutrient uptake, N and P use efficiencies, and rice grain yield in the bio-organic fertilizer (2 t ha^–1^) with 30% reduced N, 100% K, and TSP eliminated treatment. Conversely, in the farmers’ field demonstration trials, significantly (*P* < 0.05) higher partial factor productivity of N and P, plant height, tiller production, panicle number, and grain yield were obtained in the same treatment. The results of the farmers’ field demonstration trials firmly strengthen our field research findings.

After harvest of the eight crop cycle, soil nutrient contents were analyzed, and it was found that the application of bio-organic fertilizer improved soil health. Bio-organic fertilizer provided adequate mineral nutrients, organic matter, and beneficial microbial population and thus altered soil biochemical properties. It was known that soil organic carbon is an indicator of soil health by Biancalani et al. (2012), and maintaining soil quality and plant productivity requires microbial diversity and an adequate number of bacteria in the soil system (Li C. et al., 2014; Li Y. et al., 2014). The abundance of PGPB due to the application of bio-organic fertilizer was noticed after the crop harvest. The spore-forming character of *Bacillus* and *Paenibacillus* spp. (Bloemberg and Lugtenberg, 2001) was a unique character that enables survival of the added PGPB in a wide range of soil and environment. In short, the study report confirmed (4 years’) application of bio-organic fertilizer, which contained organic matter, biochar, and PGPB, improved soil organic carbon, and enriched soil with PGPB as compared with full synthetic fertilizer application.

## Conclusion

Emerging demand for food production made rice cultivation dependent on synthetic fertilizer and augmenting environmental pollution. Bio-organic fertilizer that exhibited various qualities, such as nutrient acquisition and plant growth promotion of rice, is an organic-based biofertilizer that contains RP (5%), biochar (15%), and the living cells of PGPB, mostly *Bacillus*, *Proteus*, and *Paenibacillus* spp., which were isolated from the floodplain, terrace, and saline soils. The results of 16 field experiments and 18 farmers’ demonstration trials proved that added PGPB supplemented 30% synthetic N requirement of rice production through BNF and fully complemented available P from RP by solubilization during the plant growth period. IAA production by the PGPB might have promoted plant growth and resulted in taller plants of the bio-organic added treatment. The organic matter and biochar improved soil nutrient and carbon content as well. The combined effect of living ingredients and organic matter of the bio-organic fertilizer saved 30% urea-N, eliminated 100% TSP fertilizer use in rice production, and simultaneously improved nutrient uptake, N, P use efficiencies, rice yield, and soil health.

## Data Availability Statement

The raw data supporting the conclusions of this article will be made available by the authors, without undue reservation.

## Author Contributions

UAN and JCB developed technology and wrote the manuscript. MIUS and AJ did laboratory analysis. FHK, MHRH, AI, MM, and MBH conducted experiments in BRRI research stations and attached with farmers’ field demonstration trials. MRI and MSK provided the facilities to conduct experiments in BRRI research stations. All authors contributed to the article and approved the submitted version.

## Conflict of Interest

The authors declare that the research was conducted in the absence of any commercial or financial relationships that could be construed as a potential conflict of interest.
