# Supplementary material for: Bio-Organic Fertilizer: A Green Technology to Reduce Synthetic N and P Fertilizer for Rice Production
Source: Front Plant Sci. 2021 Mar 23;12:602052. doi: 10.3389/fpls.2021.602052 (PMC8023392; doi:10.3389/fpls.2021.602052)
Supplement: Supplementary file 1 [file Data_Sheet_1.DOCX]

Accession Sample Name SPUID Organism Tax ID Strain Isolate BioProject

SAMN16084736 SSDB1 SSDB1 Paenibacillus polymyxa 1406 SSDB1 Soil PRJNA662441

SAMN16084737 SSDB2 SSDB2 Bacillus mycoides 1405 SSDB2 Soil PRJNA662441

SAMN16084738 SSDB3 SSDB3 Bacillus cereus 1396 SSDB3 Soil PRJNA662441

SAMN16084739 SSDB4 SSDB4 Bacillus sp. SSDB4 2771370 SSDB4 Soil PRJNA662441

SAMN16084740 SSDB5 SSDB5 Bacillus sp. SSDB5 2771371 SSDB5 Soil PRJNA662441

SAMN16084741 SSDB6 SSDB6 Paenibacillus sp. SSDB6 2771372 SSDB6 Soil PRJNA662441

SAMN16084742 SSDB7 SSDB7 Paenibacillus polymyxa 1406 SSDB7 Soil PRJNA662441

SAMN16084743 SSDB8 SSDB8 Bacillus sp. SSDB8 2771373 SSDB8 Soil PRJNA662441

SAMN16084744 SSDB9 SSDB9 Bacillus sp. SSDB9 2771374 SSDB9 Soil PRJNA662441

SSDB10 SSDB10 Bacillus subtilis Soil JQ820255
